# Force-Field Compensation in a Manual Tracking Task

**DOI:** 10.1371/journal.pone.0011189

**Published:** 2010-06-17

**Authors:** Valentina Squeri, Lorenzo Masia, Maura Casadio, Pietro Morasso, Elena Vergaro

**Affiliations:** 1 Italian Institute of Technology, RBCS Department, Genoa, Italy; 2 Northwestern University, Chicago, Illinois, United States of America; 3 University of Genoa, DIST Department, Genoa, Italy; The University of Western Ontario, Canada

## Abstract

This study addresses force/movement control in a dynamic “hybrid” task: the master sub-task is continuous manual tracking of a target moving along an eight-shaped Lissajous figure, with the tracking error as the primary performance index; the slave sub-task is compensation of a disturbing curl viscous field, compatibly with the primary performance index. The two sub-tasks are correlated because the lateral force the subject must exert on the eight-shape must be proportional to the longitudinal movement speed in order to perform a good tracking. The results confirm that visuo-manual tracking is characterized by an intermittent control mechanism, in agreement with previous work; the novel finding is that the overall control patterns are not altered by the presence of a large deviating force field, if compared with the undisturbed condition. It is also found that the control of interaction-forces is achieved by a combination of arm stiffness properties and direct force control, as suggested by the systematic lateral deviation of the trajectories from the nominal path and the comparison between perturbed trials and catch trials. The coordination of the two sub-tasks is quickly learnt after the activation of the deviating force field and is achieved by a combination of force and the stiffness components (about 80% vs. 20%), which is a function of the implicit accuracy of the tracking task.

## Introduction

Humans commonly make arm movements that require manipulation of objects or tools, thus interacting with the environment, like in opening a door, turning a steering wheel, and rotating a coffee mill. The constraints imposed by the environment can be hard, as in crank turning, or soft, as in pushing a movable object or keeping contact with the partner's hand while dancing. Therefore, constrained movements always involve both motion control and interaction-force components and the influence of the task is determinant in deciding which one of the two components is the leading one.

For example, in crank turning it is known [1], [2] that significant radial forces (compressing or extending the crank) are exerted despite the fact that these forces are not necessary to perform the task. In handwriting or drawing, on the contrary, force and position control are both relevant but are independent, thus leaving room to an infinite variety of writing styles. In the robotic field there are classical solutions for constrained tasks with hard constraints which are often based on hybrid control [3] or impedance control [4] using central controllers based on global knowledge of direct and inverse kinematics or dynamics. Unfortunately, this approach is not applicable to the human case because global knowledge of the environment is not generally available.

Pure force control in the absence of position control occurs in targeted force impulses in isometric conditions [5], with similar timing features (bell-shaped speed profile) of unconstrained reaching movements [6]. If the constraint becomes active in a sudden, discontinuous way, like in finger tapping movements, there is evidence that the muscle coordination patterns switch in a sudden and anticipatory manner from motion to isometric force control [7].

Also force field adaptation can be considered as a constrained hybrid task, which depends of the type of force field. For example, reaching a target in a curl viscous field [8] implements a kind of directional, dissipative constraint and it has been demonstrated that adaptation is characterized by the acquisition of an internal model of the disturbance, i.e. a force control mechanism. On the contrary, in another experimental condition (reaching movements in a divergent field) it has been found that adaptation is obtained through stiffness modulation, implemented by co-activation of antagonistic muscles [9].

In this paper we address the coordination of position and force control in a dynamic task, where the positional and force components have both a large range of variation and muscle stiffness is likely to have a role as well. The positional sub-task is continuous visuo-manual tracking of a Lissajous figure. The force sub-task is compensating a curl viscous field generated by a haptic manipulandum that pushes away the hand from the desired trajectory. The task is similar to force-field adaptation in centre-out reaching movement but with significant differences: in centre-out reaching, the positional and force control sub-tasks are uncoupled because the former one is focused on the final point of the trajectory whereas the latter is only operant in the middle portion of the movement, where the speed profile has its peak. Moreover, the performance index is well defined only for the final part of the movement. Contrarily, in tracking movements force control (the slave sub-task) must be continuously synergic with positional control (the master sub-task) during all the time in order to achieve the required tracking accuracy.

Visuo-manual tracking tasks have been studied since the early 70s [10] in order to measure human motor control and movement performance in a variety of laboratory setups and real-world skills. The mutual influence of the arm motor system and oculomotor system has been investigated in depth [11], [12] by taking into account that the visuo-motor subsystem is characterized by the interplay of two controllers, the saccadic and smooth pursuit eye system that integrate feedback and feed-forward mechanisms. It has been found that a visual memory mechanism or memory buffer, with a duration of at least one second, operates in the planning phase of visuo-manual tasks [13], thus inducing a discontinuity in the manual control signals that are indeed characterized by intermittent step-and-hold movement periods [14], [15]. In general, there is ample evidence that constrained and accurate limb movements are composed of discrete sub-movements, as part of an intermittent error detection and correction process, although an agreement on the origin of intermittency has not yet been reached: is it the consequence of neurophysiological internal constraints [16] or of specific control strategies [17]?

The experimental protocol is based on manual tracking of a target moving on a regular path (eight-shaped path), thus combining continuity with a robust spatio-temporal constraint (the so called power law that links speed and trajectory [18]). Moreover, by disturbing the subject's hand with a robot-generated force field, pushing the hand sideways with respect to the instantaneous hand velocity vector, we linked the positional sub-task (tracking the smoothly moving target) with the force sub-task (compensating the lateral deviation just enough to satisfy the tracking accuracy requirement). The force sub-task was designed in such a way to be challenging and require compensatory forces that are much greater than those employed in the unperturbed tracking task. In this way, it is possible to address two main questions: 1) Can the motor synergy, responsible of steady-state tracking behaviour, survive the disruptive action of the disturbing force field? 2) Compensation of the lateral deviations determined by the force-field is achieved by means of stiffness properties, force control or a combination of the two? These experiments are complementary with respect to other studies [19], [20] which also address force-field compensation during manual tracking: the main difference is that we focused on steady-state performance whereas the quoted authors were more interested in transient behaviour that includes intercept & tracking for a rather short time.

## Methods

### Experimental setup

The robotic workstation employed in the experiments is based on a haptic, planar manipulandum with 2 degrees of freedom (Braccio di Ferro: BdF), which has been fully described elsewhere [21]. It has a large planar workspace (80×40 cm ellipse) and a rigid structure with direct drive of two brushless motors that provide full back driveability, low intrinsic mechanical impedance, and a good isotropy index at the end-effector. The robot can measure the trajectory of the hand with high-resolution (<0.1 mm) and is smoothly impedance-controlled in order to generate continuous force fields that can range from fractions of 1 N up to 50 N. The control architecture is based on the real-time operating system RT-Lab® and includes three nested control loops: 1) an inner 16 kHz current loop; 2) an intermediate 1 kHz impedance control loop; 3) an outer 100 Hz loop for visual display and data storage.

**Figure 1:**
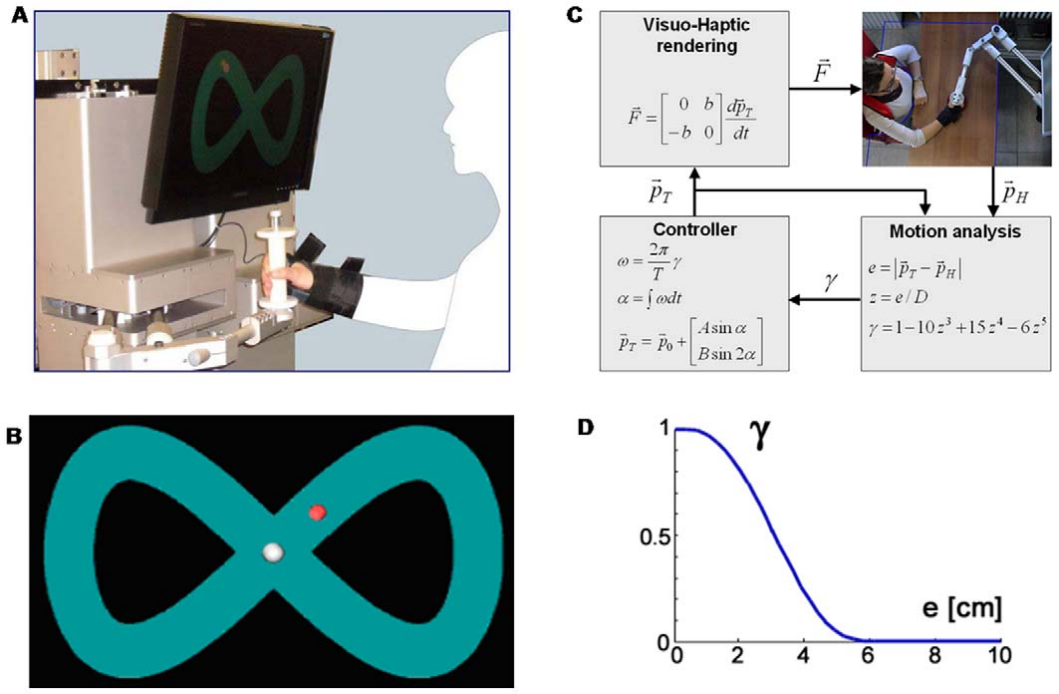
Experimental setup. **A:** Haptic manipulandum BdF used in the experiments. **B:** Dynamic visual display. The red circle identifies the position of the target and the white circle the position of the hand; both have a 2 cm diameter. The eight-shaped pathway is displayed in the background for one group of subject. For the other group the background is uniformly black. The ideal eight-shaped path is ±15.7 cm wide and ±9 cm high; total length is equal to 102 cm; the nominal circling period is 8 s. **C:** Real-time flow of information among the four main modules of the robotic manipulandum (BdF haptic manipulandum; Motion analysis and performance evaluation; Task controller; Force-field generator); 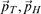: position vectors of the target and the hand, respectively; *D* = 6 cm: threshold for the tracking error *e*; 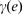: target speed modulating function; *T* = 8s: nominal circling time; *b* = 100 N/m/s: viscous coefficient of the curl field 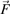. **D:** Profile of the target speed modulating gain.

The subjects sit in a chair and grasp the BdF handle (figure 1A). Their chest and wrist are restrained by means of suitable holders. A large LCD screen is positioned in front of them at a distance of about 1 m, displaying the positions of hand and target by means of two circles of different colors, with a diameter of 2 cm, on a background which can be either blank or structured, portraying the shape of the nominal trajectory.

The task is visuo-manual tracking of a simulated target 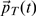 that moves according to eq. 1, describing the Lissajous figure depicted in figure 1B, with amplitude coefficients A = 15.7cm, B = 9cm (for a total length of 102 cm), and nominal circling duration *T* = 8 s:
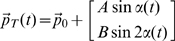
(1)The actual circling duration is longer than the nominal value because target speed was regulated as a function of the tracking error. This mechanism was introduced in order to simplify the tracking task when the force field was active. In a preliminary test we verified indeed that, without this mechanism, subjects were unable to successfully track the target during at least one complete turn, at the required level of accuracy.

The central point of the figure 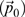 was chosen in such a way that, when the manipulandum is placed there, the elbow is flexed at an angle of about 90 deg and the shoulder at an angle of about 45 deg. The subjects were instructed to track the moving target as accurately as possible using the cursor which gives the actual position of the robot handle, in such a way that the two circles were at least touching each other. Therefore, the implicit precision of the tracking task is of the order of 2 cm.

The rotation frequency of the target (*ω = dα/dt*) is a smoothly decreasing function of the tracking error 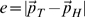, where 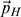 is the running position of the hand:
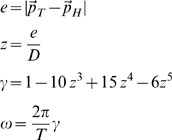
(2)
Figure 1C summarizes the real-time flow of information. The nominal circling period (T = 8 s) only occurs in the ideal situation of zero tracking error *e*. As *e* increases, the circling frequency decreases and it becomes 0 when an error threshold is reached (*e = D*). In other words, the target stops when *e≥D* and waits for the subject to re-enter the allowed error range. Thus *D* is a parameter that allows setting the degree of difficulty of the task. In the described experiments *D* = 6 cm. The angular speed modulating function *γ(e)* decreases from 1 to 0 in a smooth way (according to a minimum jerk profile) and equals 0.5 at mid range (*γ* = 0.5 for *e* = 3cm, see figure 1D).

**Figure 2:**
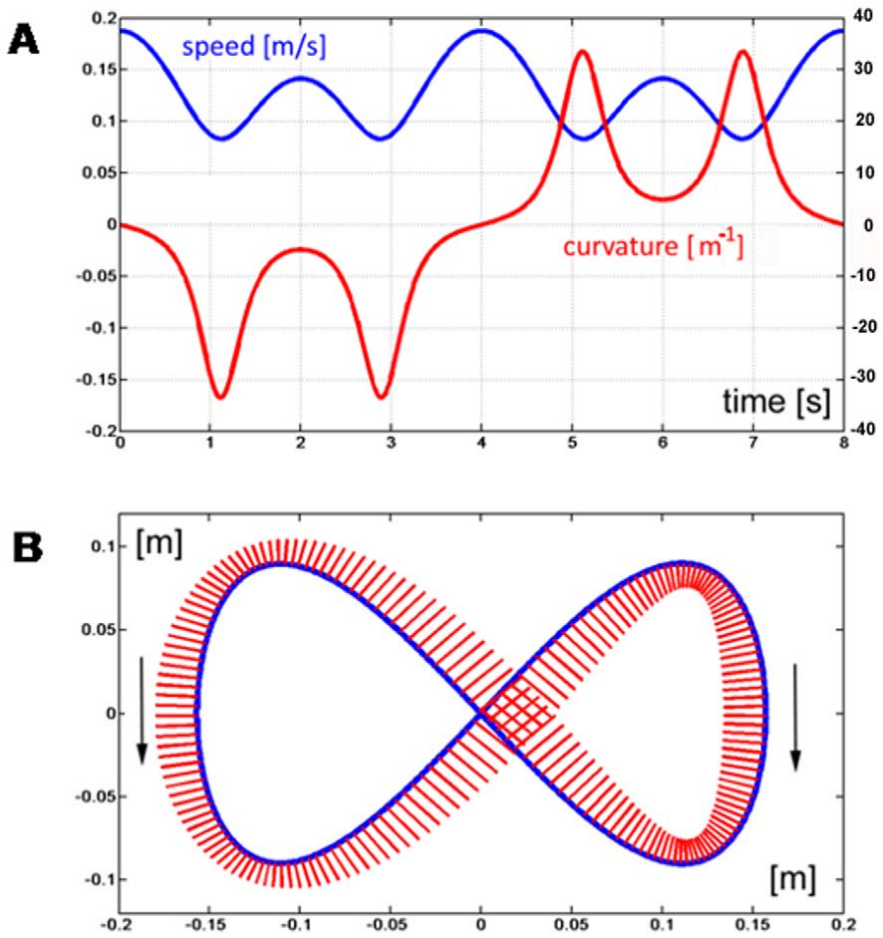
Characteristics of errorless trajectories. **A:** Speed and curvature profiles of the target in the case of ideal, errorless tracking performance. The curvature scale (in m^−1^) is divided by 200. The correlation coefficient of the two curves is equal to 0.91. **B:** Pattern of force determined by the curl field, in the case of ideal, errorless tracking performance. The force vectors, perpendicular to the eight-shaped path and directed to the right of the instantaneous velocity vector, range between 8.3N and 18.3N.

In the ideal situation, with null tracking error, each lap would be characterized by the speed/curvature profiles of figure 2A with 8s period and speed range of 8.3–18.8 cm/s.

As in the case of handwriting or drawing movements, the speed and curvature profiles of the target motion are strongly correlated [18], [22]: peak values of speed are synchronized with points of minimum of the absolute value of the curvature and vice versa. In this situation, the correlation coefficient between speed and absolute curvature is quite high (over 90%). If tracking performance decreases we may expect correlation to decrease. Curvature (the inverse of the instantaneous radius of curvature) is computed according to the following equation, which can be applied to the target and hand trajectories:
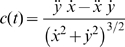
(3)The introduction in the target generation mechanism of the angular speed modulating function makes the task more challenging and rewarding because a decreasing error yields a quicker target. In this way, the mechanism adapts to the tracking performance: the curvature profile remains invariant whereas the speed profile may exhibit new peaks as a consequence of the modifications of the control patterns.

Tracking movements were recorded in two different conditions: absence and presence of a curl force field generated by the robot according to the following control law:
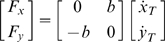
(4)The force direction is orthogonal to the tangential direction of the desired target trajectory and pushes the hand to the right, with respect to the current orientation of the velocity vector. The value of the viscous coefficient *b* was 100N/m/s, thus inducing a range of disturbance forces of 8.3–18.8 N, in the ideal case of errorless performance. This range of interaction-forces must be compared with the range of forces produced by the neuromuscular system in the undisturbed situation, under the hypothesis of errorless tracking. Inertial forces are the most relevant ones, considering that the studied movements are not affected by gravity. For simplicity, we may also restrict our attention to the acceleration-dependent forces, neglecting Coriolis forces or other effects, since we are only interested in evaluating the order of magnitude of the forces at play. Then we only need to compute the peak value of the acceleration vector over each cycle, starting from eq. 1, and we found that its magnitude does not exceed 0.3 m/s^2^. The equivalent mass of the arm at the end-effector is of the order of 1 kg [23] and also the mass of the manipulandum is of the same order of magnitude [21]. Thus the order of magnitude of the forces produced by the muscle activity during unperturbed movements is less than 1 N, which is just a fraction of the robot-generated interaction-forces. Figure 2B shows the pattern of forces generated in the ideal errorless performance: in half of the eight-shaped path the forces are directed inside and in the other half are directed outside.

### Experimental protocol and subjects

Nine young adults (age = 24±1.2y), all right-handed, participated in the experiments. Hand preference was evaluated by means of the Edinburgh Handedness questionnaire [24]. The research conforms to the ethical standards laid down in the 1964 Declaration of Helsinki that protects research subjects. Each subject signed a consent form that conforms to these guidelines.

The task assigned to them was to track the target shown on the screen as accurately as possible, in such a way that the target circle and the hand circle were at least touching each other. Since the two circles have a 2 cm diameter, the spatial resolution of the task was implicitly set to about 2 cm.

The subjects were randomly divided into two groups, according to the type of visual background on the computer screen: in one group (G1: 5 subjects) the background portrayed the Lissajous figure, as shown in figure 1B; in the other group (G2: 4 subjects) the background was blank. Both groups had vision of the hand and target positions on the screen. The rationale of this detail of the protocol was to check whether the full vision of the pathway could have an effect on the tracking/adaptation performance.

Experiments were organized in target-sets. Each set consisted of 30 proper turns, i.e. turns that were completed in less than 12 s (50% longer than the nominal duration) and possibly failed turns (i.e. turns with a duration exceeding 12 s). For the target-sets in which the force field was activated there were also 6 catch trials, i.e. randomly selected turns in which the force field was switched off for 0.5 s, when the target crossed the central point of the trajectory with a tracking error less than 2 cm. Half of these catch trials were related to rightward movements and the other half to leftward movements.

The protocol comprises the following phases: familiarization phase (2 target-sets, FA1 and FA2, with null field); field adaptation phase (2 target-sets, FF1 and FF2, with continuously activated force-field); wash-out phase (1 target-set, WO).

### Data analysis

During the experiments we recorded the time course of the trajectory of the hand and the target, sampled at 100 Hz. The x and y components were smoothed with a 6th order Savitzky-Golay filter (window size 170 ms, equivalent to a cut-off frequency 11 Hz), which was also used to estimate the first two time derivatives. From these data, we estimated two groups of parameters that characterized, respectively, 1) tracking performance and 2) force control performance. As regards the first group we considered the following parameters: *DUR*, *FE*, *NP*, *δ*, *CC*.

#### *DUR*: mean duration of each turn

Its value must be compared with the nominal value of 8 s, which characterizes errorless tracking.

#### *δ*: Tracking error

**Figure 3:**
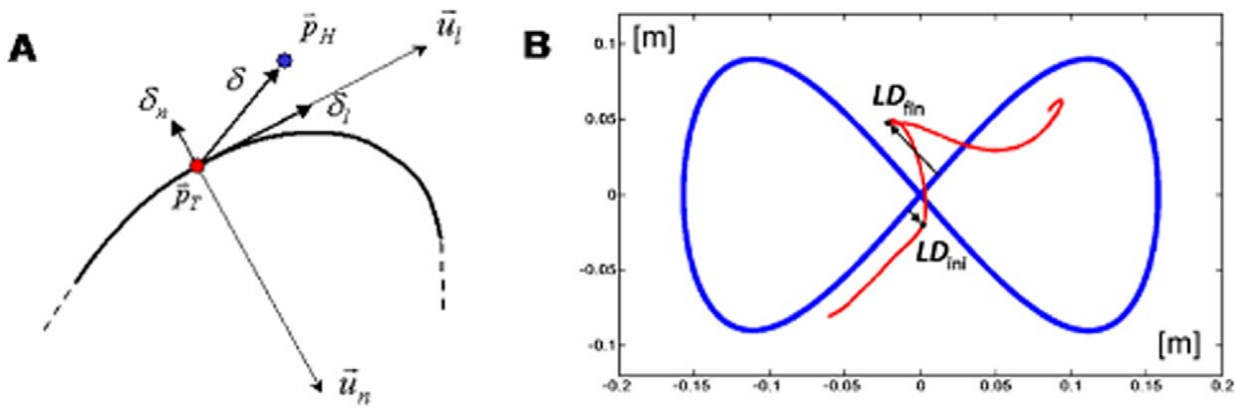
Tracking error and lateral deviation. **A:** Decomposition of the tracking error into a longitudinal and normal component. 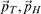: position vectors of the target and the hand, respectively; 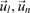: longitudinal and normal unit-vectors, respectively. **B:** Characterization of a catch trial in terms of the initial and final values of the lateral deviation (*LD_ini_ and LD_fin_*, respectively).

It is the mean value of the distance between the target and the hand over a turn and it is decomposed into two components: the longitudinal component (*δ_l_*) and the normal or lateral component (*δ_n_*), see figure 3A. While *δ* is always positive, *δ_l_* and *δ_n_* are signed quantities: *δ_l_* is positive if the hand is ahead of the target and *δ_n_* is positive if it is on the right. The two error components are computed as follows, after having defined the running unit-vectors 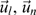 (longitudinal and normal unit-vectors, respectively):
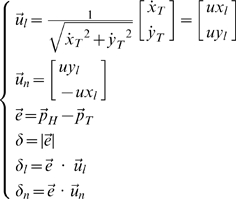
(5)


#### *FE*: figural error

It is defined as distance measurement between the ideal trajectory generated by the target and the observed trajectory [25]. It is intended to express the difference in the shapes of the respective paths and is insensitive to differences in speed between the end-effector and the target. Assuming that the two time series *A* = [*A_1_*,*A_2_…A_m_*] and *B* = [*B_1_*,*B_2_…B_n_*] represent the trajectory of the target and the hand, respectively, *FE* is computed as follows:
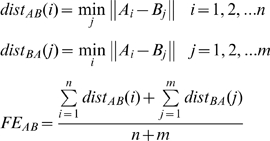
(6)


#### *NP*: average number of sub-movements per turn

Each sub-movement is identified by a peak in the velocity profile. Following previous works [18], [21] speed pulses were identified by finding the local minima in the speed profile and extracting the speed profile between two consecutive minima. It measures the degree of segmentation of the tracking process. We measured it from the hand's speed profile. This number should be compared with the four expected peaks in the ideal, errorless performance (see figure 2A).

#### *CC*: Correlation coefficient of the hand's speed and curvature profiles

The measured (absolute) values should be compared with the value that characterizes errorless performance (91%).

As regards the overall characterization of the control of the interaction-forces, we focused the analysis on the lateral deviation (*LD*, minimum instantaneous distance between the arm position and the path drawn by the target). *LD* is similar but not equal to the lateral component of the tracking error *δ_n_*, in particular during the catch trials when the force field was temporarily removed. In the undisturbed condition, we may expect this error, in agreement with the literature on visuo-manual tracking, to be small and unbiased. On the contrary, in the force field trials it might be that the error is biased in the direction of the disturbing force *F_D_* that, as illustrated by figure 2B, always pushes the hand to the right of the instantaneous velocity vector of the target. The amount of this error is a function of the force-control compensation mechanism. In general, *F_D_* will be counterbalanced by a commanded force *F_C_* and a force due to the mechanical impedance of the arm in the lateral direction. Moreover, we made the hypothesis, to be verified in the experiments, that *LD* is small and slowly changing, allowing us to make a first-order approximation that reduces the mechanical impedance to its elastic component, i.e. the stiffness *K*. Thus we can write the following equation, which characterizes instant by instant the equilibrium in the direction orthogonal to the eight-shaped path:
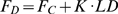
(7)An underlying, simplifying assumption is that the mechanical impedance remains constant during the catch trials. Of course this is not true but we think that it still can be taken as a first order approximation, applicable to the context of this study. In this equation *F_D_* is known but we are not able to differentiate between the relative contribution of the two components (*F_C_* and 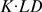) only by looking at the evolution of *LD*. A possibility is to analyse the behaviour of the catch trials, which is schematised in figure 3B. Let us denote with *LD_ini_* and *LD_fin_*, the lateral deviations of the hand at the beginning and the end, respectively, of a catch trial. According to the chosen sign convention *LD_ini_*>0 and *LD_fin_*<0. One should also consider that during catch trials the force field is suddenly switched off always in the same position (the crossing point of the Lissajous figure). Thus we can assume that the amplitude of the force impulse is approximately constant. In particular, let us re-write eq. 7 for two different positions and time instants: before the beginning of the inactivation interval (*t_0_*) and before the end (*t_F_*):
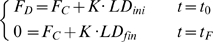
(8)The first equation expresses the balance between the force field disturbance, the commanded force, due to voluntary force control, and the force due to end-point stiffness. In the second equation, which is related to the final sample of the inactivation impulse, *F_D_* = 0 by definition. From these equations we can recover the two unknowns, the stiffness and the commanded force, from the measured variables (*F_D_*, *LD_ini_* and *LD_fin_*):
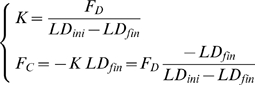
(9)Summing up, we defined the following indicators related to the control of the interaction-forces: *PAC* and *FMC*.

#### *PAC:* Proportion of active force compensation

This indicator is intended to estimate the percentage of compensation of the force-field disturbance which can be attributed to voluntary force control, in relation with the total disturbance.
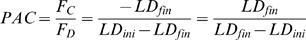
(10)With the conventions described above, *LD_fin_* is expected to be a positive quantity whereas *LD_ini_* is expected to be negative. Thus *PAC* should be positive and smaller than 1.

#### *FMC*: Force Movement Correlation

We measured the correlation coefficient between the speed profile of the tracking movements and the lateral deviation, for each lap. The speed profile is characteristic of the positional component of the investigated task while the lateral deviation is determined by the interaction-force controller. Thus, this coefficient provides a measure of the degree of independence of the two control actions.

### Statistical analysis

The analysis of the learning process was focused on the tracking error *δ*. For each subject we computed the average along each turn, taken as the basic computational unit. The evolution of this parameter for the whole population of subjects in the different phases of the protocol (FA, FF, and WO) was fitted by means of exponential functions in order to evaluate the time constant of learning/adaptation
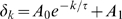
(11)The parameter *A_1_* is the asymptotic magnitude of the tracking error *δ* (after an infinite number of trials); *A_0_* represents the variation of this error between the first lap and an infinite number of laps (thus indirectly specifying that the initial error is *A_ini_ = A_0_+A_1_*); *k* is the lap counter; *τ* is the decay time constant, expressed in number of laps or turns.

The analysis of steady-state performance (we used the program STATISTICA 7, Stat Italia srl) focused on the comparison between FA2 vs. FF2. As these phases included a fixed number of proper turns and a variable number of failed turns, for each performance parameter we ran variance components analysis that allows to compare factors with different number of cases. We set *SUBJECTS* as random factors and *DURATION* of movement as covariate. This latter adjustment reduces the variation caused by different lengths of turns and as consequence, it allowed us to consider both proper and failed turns. Moreover we had two fixed factors: *EXPERIMENT* (G1, G2) and *PHASE* (FA2, FF2).

As regard the *PAC* and *FMC* index, we run a two-ways ANOVA with factors *EXPERIMENT* (G1, G2), *PHASES* (FF1, FF2).

## Results

### Baseline performance in the null-field condition

**Figure 4:**
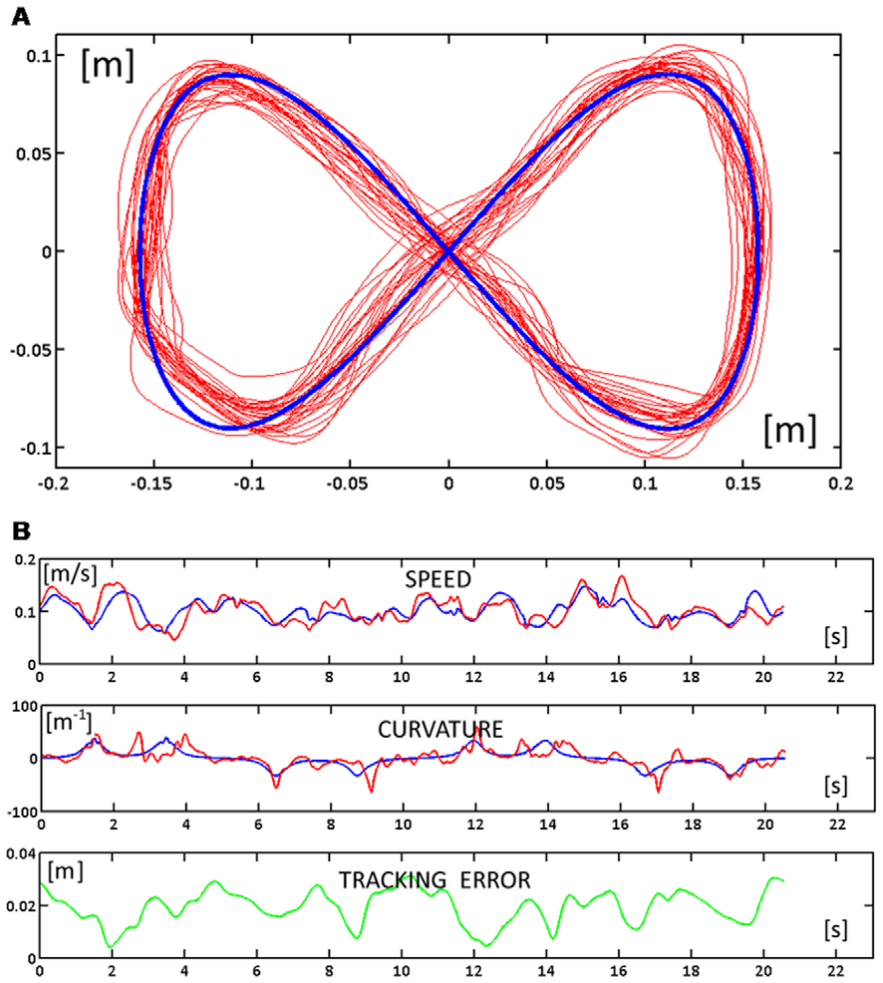
Response patterns of one subject at the end of the familiarization phase during two consecutive turns (FA2). **A:** Trajectories of the subject (red) and trajectory of the target (blue) for the whole phase. **B:** Time course of the speed (blue for the target and red for the hand), curvature (blue for the target and red for the hand), and tracking error (green).

All subjects quickly adapted to the experimental setup and protocol and in the late part of the familiarization phase the recorded patterns became quite consistent. Figure 4 shows a typical behavior in the spatial and time domain for a single subject, in the middle part of session FA2, i.e. near the end of the familiarization phase. The tracking performance parameters for the whole population are as follows (values are means±SE):

- *DUR* = 9.5±0.2 s (duration of each turn): it is slightly longer than the nominal duration for errorless performance (8 s).
- *NP* = 15.2±0.1 (number of peaks in the speed profile): it is about four times the nominal number for an errorless performance, suggesting an average intermittent control frequency of about 1.6 Hz. A similar value (1.6±0.21 Hz) was found by carrying out a frequency analysis of the speed profiles and detecting the peak values.
- *δ* = 16.3±1.1 mm (total tracking error), decomposed into a longitudinal component *δ_l_* = −11.9±1.2 mm (the minus sign means that the hand is slightly lagging the target) and a lateral or normal component *δ_n_* = −1.0±0.4 mm, which is statistically equivalent to a null lateral displacement, as expected. In summary, the subjects can track the target with a little lag (compatible with the spatial resolution of the task) and negligible lateral displacement.
- *FE* = 6.2±6.2 mm (figural error): it is small and compatible with the tracking error considered above.
- *CC* = 51.9±0.7% (correlation coefficient between the speed and the curvature profile): it is lower than the ideal one (91%) but over 50%.

**Figure 5:**
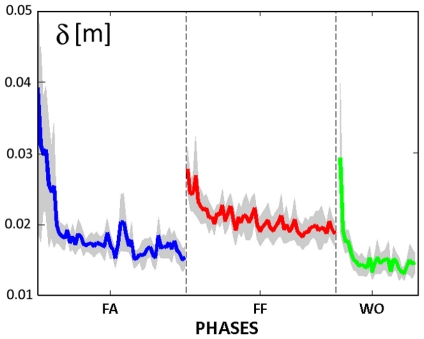
Learning curve of the tracking error δ for the whole population of subjects: mean ± standard error. FA: familiarization phase (60 turns); FF: force-field phase (60 turns); WO: wash-out phase (30 turns).

The speed of the familiarization phase is depicted in the leftmost graph of figure 5, which shows the time course of the tracking error *δ* during the whole phase. The graph was fitted with an exponential function, yielding a familiarization time constant *τ_fam_* = 4.6 laps (r^2^ = 0.916).

### Learning during force field adaptation

**Figure 6:**
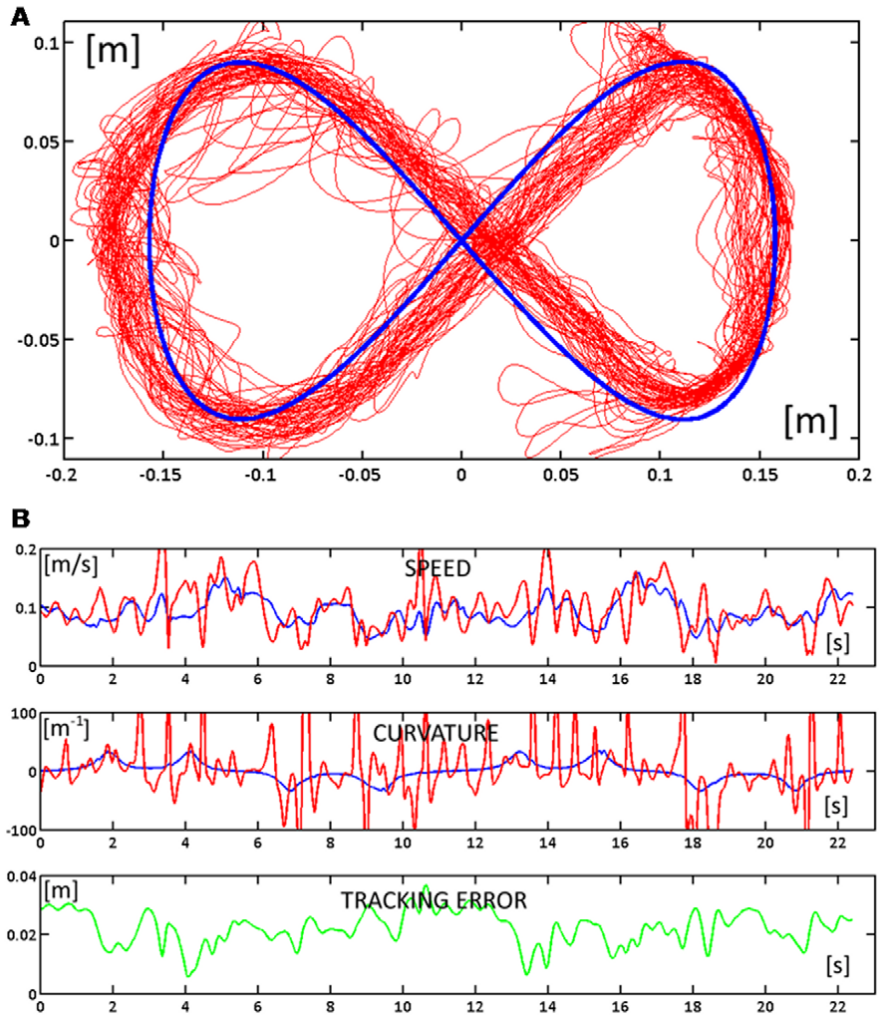
Response patterns at the beginning of the force field phase during two consecutive turns (FF1). **A:** Trajectories of the subject (red) and trajectory of the target (blue) for the whole phase. **B:** Time course of the speed (blue for the target and red for the hand), curvature (blue for the target and red for the hand), and tracking error (green).

**Figure 7:**
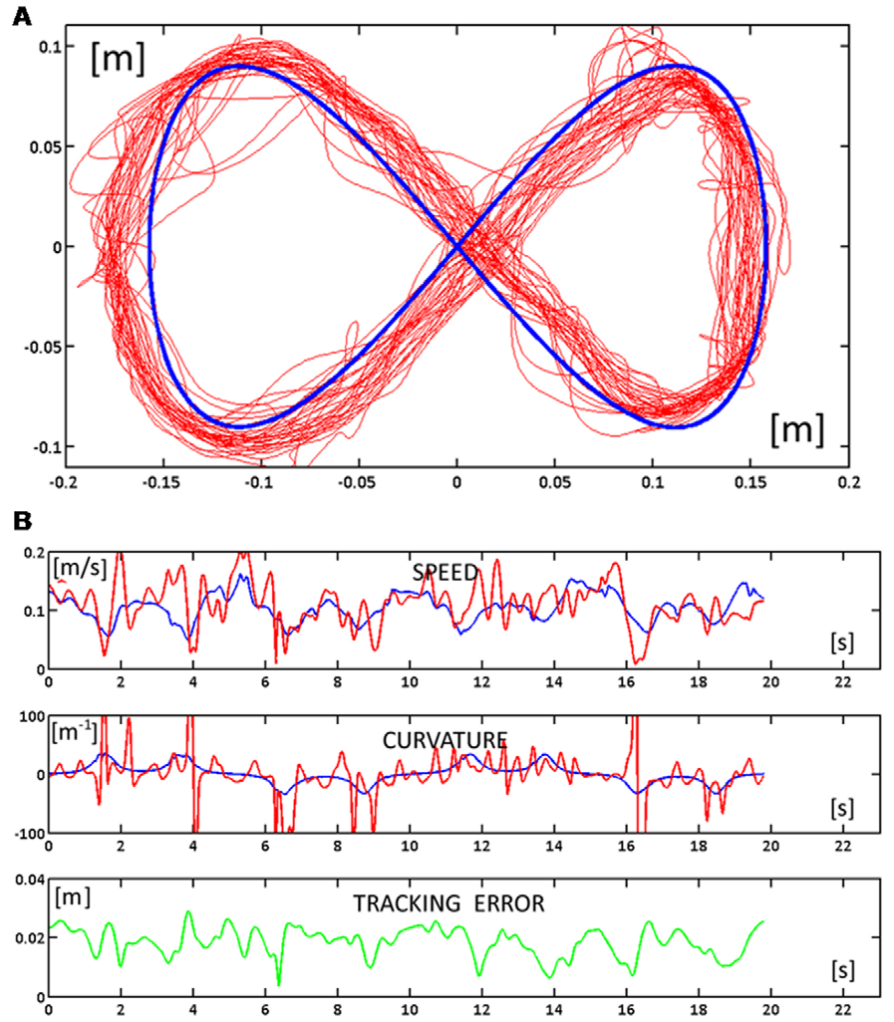
Response patterns at the end of the force field phase during two consecutive turns (FF2). **A:** Trajectories of the subject (red) and trajectory of the target (blue) for the whole phase. **B:** Time course of the speed (blue for the target and red for the hand), curvature (blue for the target and red for the hand), and tracking error (green).

When the force field is activated, the subject's ability to track the moving target is initially disrupted, probably due to an inability to predict abrupt changes in the direction of the perturbing force. This is visible, for example, in figure 6A which displays the hand trajectories during the whole FF1 phase. Apart from such occasional episodes in the very initial part of the first force field exposure, performed trajectories were clearly coherent with the accuracy requirements of the tracking task during most of the time, as shown in figure 6B, which displays the time course of speed, curvature, and tracking error during a fragment in the early part of FF1 (laps 6 and 7). The performance in the FF2 phase is illustrated in figure 7: the top panel (figure 7A) displays the hand trajectories during the whole phase and the bottom panel (figure 7B) the time course of the relevant variables during two consecutive laps in the middle of the phase.

The speed of the learning process can be appreciated by looking at figure 5, which displays, for the whole population of subjects, the evolution of the tracking error *δ* averaged on each lap of the Lissajous figure. The statistical analysis shows that, after the introduction of the force field, the increase of *δ* is significant (F = 20.816, p = 0.001660) but it tends to decrease during the whole force-field phase, in good agreement with the exponential fitting: time constant *τ_ff_* = 9.43 laps (r^2^ = 0.749). Moreover, there is no significant difference of the tracking error *δ* between FA2 and FF2 as well as between FA2 and WO, demonstrating that the performance of the subjects normalized over the course of the task.

### Performance at the end of force-field adaptation

**Figure 8:**
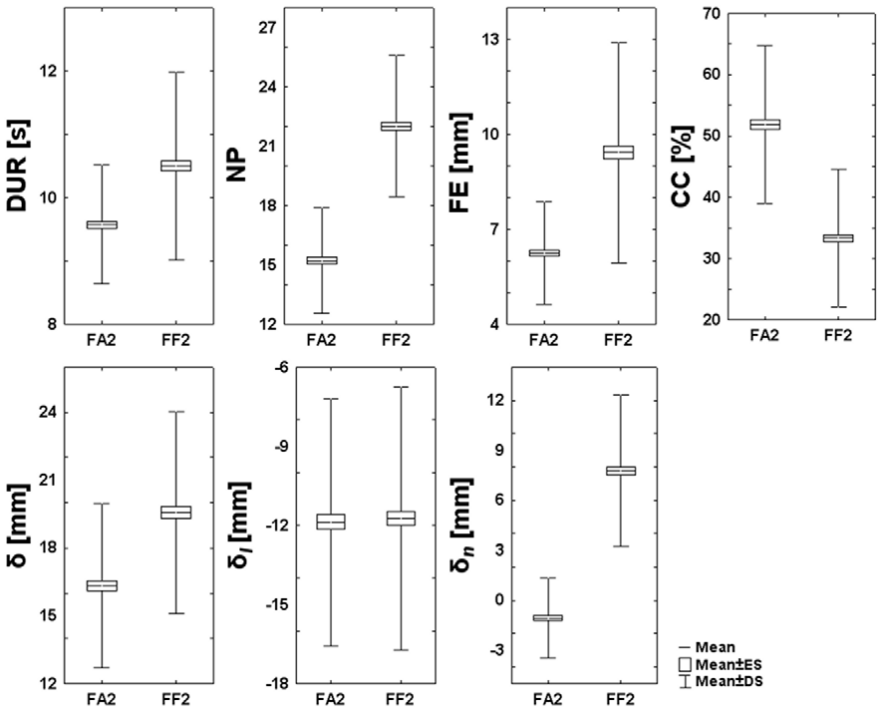
Comparison of the tracking performance indicators between the end of the familiarization phase (FA2) and the end of the force field phase (FF2). The data are averaged over the whole population. Each box shows the mean value (central line) and the mean±SE (lower and upper line). The lines extending from each end of the box represent the mean±SD. DUR (duration of each turn); NP (number of peaks for each turn of the hand speed profile); FE (figural error); CC (correlation coefficient between speed and curvature of the hand); 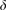 (tracking error); 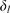 (longitudinal component of the tracking error: negative means that the hand is lagging); 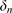 (lateral or normal component of the tracking error: negative means that the hand is deviated on the right of the target direction). *NP*, *FE*, CC and 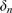 exhibit significant differences between FA2 and FF2 at the p = 0.00000, p = 0.008340, p = 0.000028 and p = 0.000025 significance level, respectively.

How well did the subjects succeed to master the hybrid force-movement task? We should remark that it is a rather fatiguing task. The lateral pushing force oscillates between 8 and 18 N, with an average value of about 12 N, and it must be maintained during a whole target-set that usually lasts more than 5 min. In spite of this, we can see from figures 6 and 7 that the anti-correlation between the curvature and speed profiles is basically preserved, although the trajectory segmentation is higher than in the unperturbed phase, as demonstrated by the higher number of speed pulses during exposure to the force field. However, the most remarkable difference, visible in figures 6A and 7A, is that the lateral tracking error is systematically biased to the right of the current movement direction, i.e. in the direction of the force field. More specifically, there is the following change of the tracking performance parameters from FA2 to FF2 (see also figure 8 for a summary):

- *DUR* = 10.2±0.2 s is slightly increased from the value in FA2 (9.5±0.2 s) (F = 8.79985, p = 0.017915).
- *NP* = 21.7±0.1 is significantly increased from 15.2±0.1 (F = 344.56, p = 0.00000).
- *δ* = 18.7±1.6 mm is basically the same of FA2 (16.3±1.1 mm) and this means that the subjects can satisfy the task requirements even in presence of the disturbing force field. Also the longitudinal component of the tracking error is basically unchanged (−11.4±1.4 mm vs. −11.9±1.2 mm). The only sharp difference is in the lateral component of the tracking error, which jumps from a virtually null value (−1.0±0.4 mm) to 7.1±1.5 mm (F = 88.96, p = 0.00002). This systematic bias, in the direction of the force field, shifts the generated trajectories “inside” the nominal Lissajous shape in the rightward part which is run clockwise, and “outside” the shape, in the leftward path which is run counter-clockwise.
- *FE* = 8.7±1.1 mm (figural error): there is a rather small but significant increase (from 6.2±6.2 mm; F = 12.72, p = 0.00834) which a consequence of the higher segmentation and systematic bias of the lateral component of the tracking error.
- *CC* = 33.6±0.6% (correlation coefficient between the speed and he curvature profiles) is significantly decreased from the value in FA2 (51.9±0.7% F = 81.28, p = 0.000028) as could expected from the increased segmentation rate.

Moreover, the statistical analysis of these parameters didn't show statistical significant effects as regards the *EXPERIMENT* factor, suggesting that the specific type of visual feedback is irrelevant for the investigated task and confirming the robustness of the coordination between the two control sub-tasks.

All together, the data support the idea that, in spite of the difficulty of this hybrid task, the subjects can quickly recover the required performance, basically preserving most spatio-temporal features of the tracking movements as in the unperturbed condition.

We also found that in the wash-out phase the subjects quickly recovered the tracking performance of the familiarization phase, with a short time constant of the exponential fit *τ_wo_* = 1.31 laps (r^2^ = 0.925). Moreover, all indexes didn't show any significant difference between the FA2 and WO phases.

**Figure 9:**
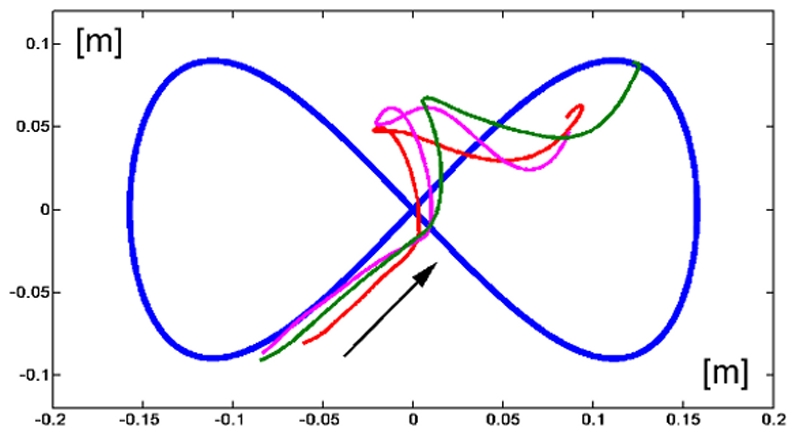
Response patterns during rightward catch trials of FF2 phase.

As regards the force control component of the task, we should consider, at the same time, the systematic bias of the lateral deviation and the behaviour during the catch trials (figure 9). The lateral component of the tracking error has a much smaller variation coefficient (43.8%) than the number of peaks of the speed profile of the tracking movement (77.3%) which determines the time course of the disturbing force; this suggests that the force compensation/control mechanism is rather efficient and well coordinated with the tracking control mechanism. The catch trials, as illustrated by the examples of figure 9, are characterized by the fact that in all cases the hand trace jumped from the right to the left of the eight-shaped path. The following values (mean±SE) of the force control indicators were computed for FF1 and FF2:

- *PAC* = 79.8±2.9% and 81.0±3.5% (proportion of active force compensation). This means that the subjects were able to supply sideways forces which were sufficient to satisfy the overall task requirement (keeping the error below 2 cm on average) but insufficient to stay onto the nominal path. The missing 20% of sideways force was provided automatically by the intrinsic muscle stiffness of the contracting muscles.
- *FMC* = 7.6±2.0% and 7.1±1.4% (force-movement correlation). The small size of this correlation, taken together with the high value of the *PAC* index and the small value of the variation coefficient of the lateral error, suggests that the force controller was indeed quite efficient in modulating the profile of the controlled force in such a way to stabilize the lateral component of the tracking error.

The statistical analysis of these parameters didn't show statistical significant effects as regards the evaluated factors (*EXPERIMENT*, *PHASES*).

Let us consider again equation 7 that characterizes the sideways control force: 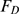 is not constant but is modulated by the speed of the tracking movement and it must be counteracted by the controlled force and the stiffness-related force. It is known that muscle stiffness increases with the exerted force in an approximately linear way [26]. Since *LD* remains approximately constant over a turn, it follows that 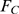 is controlled in such a way to replicate the profile of 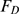, although with a gain smaller than one (approximately 0.8 according to the estimated value of the *PAC* parameter). In other words, the bias of the lateral component of the tracking error cannot be interpreted as “incomplete learning” because the overall tracking error remains inside the required tolerance.

Within the limits of validity of the linearized equation 7 and assuming that the stiffness *K* is approximately a linear function of the commanded force 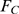, then there is a linear relationship between the lateral displacement *LD* and the ratio between the force field intensity and the intensity of the commanded force 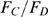. This means that after the accuracy of the task is given (by specifying *LD*), then the relative amplitude of the commanded force with respect to the force field is uniquely determined: for the task investigated in this study 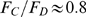 for 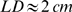.

## Discussion

**Figure 10:**
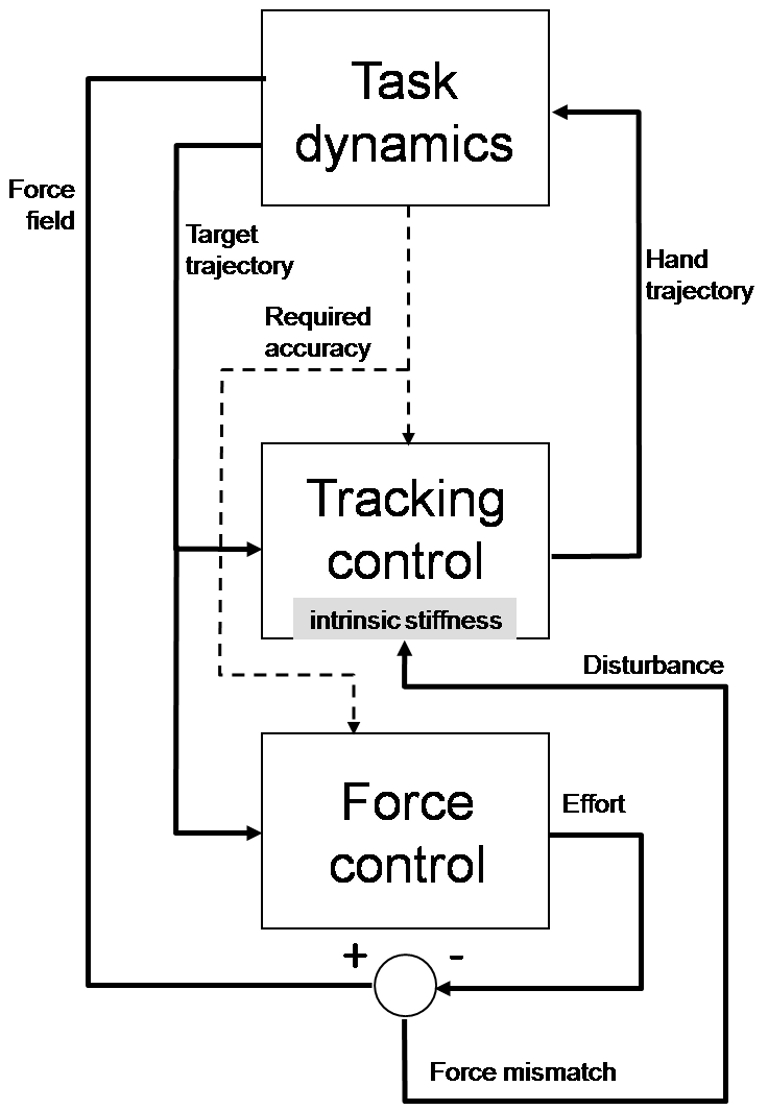
Block diagram of the hybrid control task.

In hybrid manipulation tasks that involve a movement and a force sub-task we may have different relationships between the sub-tasks. They can be independent like in handwriting, when one writes on a hard surface, or can be characterized by a master-slave relationship, like in the experiments described in the present study. In this case, the master sub-task is the tracking movement, with the tracking error as the primary performance index. The force sub-task is a slave process because it must produce a lateral push that depends on the speed of the movement. The accuracy of the force compensation is determined by the required accuracy of the tracking task. The block diagram of figure 10 summarizes the flow of information/command signals.

The master tracking-control process generates a sequence of control commands that allow the hand trajectory to track the target with the required accuracy. Ongoing disturbances, due to unaccounted dynamic effects, are counteracted by the intrinsic stiffness of the activated muscles. In our experiments, the disturbance is the mismatch between the force field generated by the task dynamics and the commanded force. The results show that, although challenging, the subjects succeed to master this hybrid task rather quickly without changing the overall structure of the master control process. In particular, the subjects could consistently tolerate a level of mismatch between the disturbing field and the commanded force that allows the intrinsic stiffness to achieve an overall balance and keep the tracking error within the required accuracy. In other words, given the basic task constraint on the required accuracy and the biomechanical properties of the intrinsic muscle stiffness, the effort produced by the subjects in the force control sub-task was sufficient to cover 80% of the required force, the remaining 20% being provided by the muscle properties. It is quite likely that such percentage depends on the required accuracy. This is the topic of future experiments.

The reported experiments help to shed some light on the vexing question about the relationship between the two groups of processes that must simultaneously operate when we manipulate an object, in the context of a specific task: 1) control of the contact forces exerted on the object and 2) control of the movements of our hand. Are the two processes combined in a unitary computational mechanism or are motions and contact forces represented and controlled by separate neural mechanism? The best known example of unitary theory for the control of hybrid tasks can be derived from the equilibrium point hypothesis (EPH) [27]–[29], which views posture as the biomechanical consequence of the interaction among the spring-like behavior of muscles and reflexes and movement as the transient from an equilibrium state to another. Although EPH was originally formulated for explaining unconstrained movements, it was later extended to the control of static contact forces in constrained movements by assuming that the same equilibrium position that guides the execution of free movements could be shifted beyond the constraint, inducing a contact force as a consequence of such shift and the intrinsic muscle stiffness [30]. This is obviously a tempting idea for its apparent simplicity, but its biological plausibility can be challenged from different viewpoints: for example, it is not clear at all how the brain could possibly compute the equilibrium points, hidden inside the object's surface, without relying on perceivable object's features. Moreover, the already mentioned experiments by Venkadesan & Valero-Cuevas [7] and by Chib et al. [31] clearly show that in hybrid tasks with a hard constraint, position and force control are dissociated. However, our experiments suggest that such dissociation may depend on the specific characteristics of the task, with particular emphasis on the compliance of the constraint. In our case the constraint is compliant, consisting of a kind of thick, soft tissue on top of a hard backing. The task is to track a target that moves on the backing surface while pushing on the tissue surface just enough to achieve the required accuracy. The recorded patterns are quite consistent with an EPH framework but without relying on some mysterious process that locates the moving equilibrium point somewhere beyond the reference surface. In the computation of the PAC indicator we assumed indeed that the equilibrium point slides on the surface of the constraint. Therefore, the force sub-task integrates a force control and stiffness control component, taking advantage of the “affordance” provided by the mechanical properties of the contracting muscles but without requiring any specific and independent modulation of stiffness. We suggest that this may explain the velocity of the learning process of our paradigm in comparison, for example, with the long time required to learn the complex coactivation patterns that are required in the adaptation to a divergent field [9].

The fact that force and movement control cannot be completely independent processes in general is suggested by experiments on delayed stiffness [32] that prompted the authors to propose a unifying computational model of stiffness perception based on an active process that combines the concurrent operations of a force and of a position control system. Moreover, other studies [33], [34] prove that position and force feedback are flexibly matched to position and force tasks by appropriately tuning the weights of the sensory channels.

In general we believe that the study of hybrid force/position control paradigms is far from having exhausted the whole range of really relevant questions. There is still the need of a well articulated set of experiments that attempt to uncover the complex set of dependencies among task requirements, physical object properties, neuromuscular features, multimodal sensory feedback, control mechanisms, and motor cognitive processes. Without such multifaceted understanding it is easy to draw conclusions that may be only valid in a narrow context and contradict each other in a wider view. Because human motion is much more than what meets the human eye.
